# An efficient serialized hardware implementation of the ASCON algorithm

**DOI:** 10.1038/s41598-026-63223-6

**Published:** 2026-07-24

**Authors:** Hasan Abdel Aziz Mohamed, Mariam Taher, Hossam Moetaz Shousha, Ziad Mohsen, Mostafa M. Abdelrazik, Yehea Ismail, Ahmed Saeed

**Affiliations:** 1 Center of Nanoelectronics and Devices, The American University in Cairo, Cairo, Egypt; 2 Department of Electronics and Communications, Cairo University, Giza, Egypt; 3 Electrical Engineering Department, Future University in Egypt, Cairo, Egypt

**Keywords:** ASIC, IoT, LWC, ASCON, Sponge construction, FPGA, Electrical and electronic engineering, Computer science

## Abstract

The era of the Internet of Things (IoT) introduces new technologies alongside challenges in hardware and data transfer security. This work presents the hardware implementation of the Lightweight Cryptography (LWC) algorithm ASCON-128, specifically targeting IoT applications and edge devices with stringent power and area constraints. The proposed design utilizes a serialized hardware architecture that minimizes logic usage, resulting in a compact area and low power consumption. It maintains adequate throughput through a sponge-based construction in authenticated mode. On FPGA, the design operates on the ultra-low-power platform, Lattice iCE40, achieving maximum frequency (Fmax) of 228.8 MHz with only 582 Lookup Tables (LUTs), 418 flip-flops, and 2.28 mW at 13.7 MHz. The Application-Specific Integrated Circuit (ASIC) implementation using 16 nm TSMC FinFET technology achieves a power consumption of 0.254 mW and an area of 1.341 kilo gate-equivalents (kGE), delivering an energy efficiency of 0.106 nJ/bit and a throughput of 2.381 Mbps at 100 MHz. These results highlight the effectiveness of the proposed ASCON-128 architecture for secure, low-power IoT and RFID applications.

## Introduction

The rise of the Internet of Things (IoT) is becoming increasingly evident, with integration expected in nearly every aspect of daily life^1^, alongside the widespread adoption of Radio-Frequency Identification (RFID)^2^. This emerging technology highlights the critical need for security across communication nodes and subsystems to protect data from unauthorized access and side-channel attacks within the network^3^. However, IoT devices are highly constrained in power and area, requiring high throughput efficiency. Traditional algorithms such as Advanced Encryption Standard (AES) and Data Encryption Standard (DES), commonly used for hardware security, are unsuitable for IoT applications due to their high power and area consumption. This overhead primarily results from complex operations, including key expansion and the MixColumns step in AES. Consequently, LWC has been developed to address these challenges, offering low power and area consumption while maintaining the required level of security^4^. To promote secure solutions for small devices, the National Institute of Standards and Technology (NIST) launched a global competition focused on Lightweight Cryptography. The Competition for Authenticated Encryption: Security, Applicability, and Robustness (CAESAR) selected the ASCON family, which provides two cryptographic functionalities: Authenticated Encryption with Associated Data (AEAD) and a hashing mode^5^. The main advantage of ASCON lies in its innovative permutation design and efficient authenticated encryption mode. This architecture offers resilience against implementation errors and attacks while enabling efficient and secure deployment^6^. As a result, ASCON advanced from the initial 57 submissions in the CAESAR competition to reach the final public evaluation.

Research on ASCON spans various areas, including permutation modifications, mathematical analysis, and the investigation of potential vulnerabilities such as side-channel attacks and hardware faults^7–9^. The limited speed of software implementations in critical applications necessitates a shift toward hardware implementations to achieve adequate performance with ultra-low power consumption. The proposed design targets applications such as RFID, Wireless Sensor Nodes (WSN), and embedded systems. RFID tags, which rely on authenticated encryption, generate power passively from the reader’s field. However, this results in a limited operational range due to power constraints, as well as increased production costs for miniaturized tags.

**Fig. 1:**
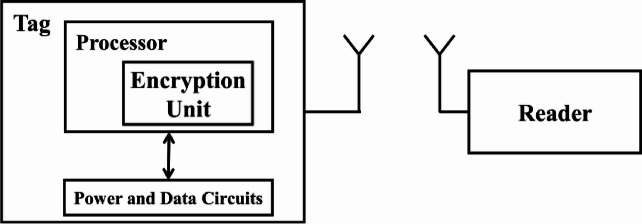
RFID system explanation.

Both embedded systems and wireless sensor nodes require microprocessors, which introduce challenges in achieving adequate security in strict areas and power constraints. To address this, the ASCON implementation is designed to minimize power and area consumption while maintaining acceptable throughput (TP). The first hardware implementation of ASCON was introduced in^10^. The initial architecture targeted applications such as RFID tags (as shown in Fig. 1), wireless sensor nodes, and embedded systems. It was implemented using the UMC 90 nm technology node. It presented three implementations of ASCON, each tailored to achieve specific design goals. Additionally, a side-channel protected design was proposed using a threshold implementation scheme, though this increased the cell area. While the ASCON-fast implementation with one unrolled round showed impressive results in terms of area and performance, it consumed 529 µW at 100 MHz, nearly double the power consumption of our proposed design at the same clock frequency. In the following years, research continued to expand on this work^11^, exploring various approaches to hardware implementation for authenticated encryption (AE) schemes. These studies focused on designs optimized for high throughput, particularly in IoT applications. The research demonstrated that processing multiple bits per clock cycle significantly affects power consumption, area, and the throughput-to-area ratio, especially in Field-Programmable Logic Array (FPGA) implementations. Their hardware implementation relied on three unrolled, round-based, and serialized strategies. The serialized implementation aims to process multiple bits per cycle to increase throughput. It was found that processing 2-bit per cycle increases the TP to 230.8% compared to 2-bit per cycle, with only a 36.8% increase in area. In a round-based implementation, they tried to improve TP in an FPGA by implementing several possible permutations in one clock cycle, but it has more area than the serialized implementation. In addition, they implemented the first unrolled ASCON in an FPGA with TP of 766.96Mbps and 1389.2 Mbps for ASCON-128 and ASCON 128-a, respectively.

Additionally, some studies aim to enhance flexibility and energy efficiency^12^. The proposed design accommodates a range of IoT applications by implementing authenticated ciphers (encryption and decryption processes) and hash functions in a compact six-mode configuration. Evaluations using 28/32nm technology revealed a high throughput, achieving 29% less power consumption and operating at frequencies over 667 MHz. This design has also been implemented using SkyWater130nm technology. Further research examined the implementation of side-channel attack mitigation and fault injection protection mechanisms for ASCON^13^. This work employed threshold and triplication/majority techniques and was applied to ASIC technology (STM 130 nm node) and Xilinx-based Kintex-7 FPGA. They presented four implementations of the ASCON: Unprotected ASCON (encryption + tag generation, decryption + tag verification; and hashing), Side-channel attack resistant ASCON using threshold implementation, fault attack resistant ASCON using triplication/majority, and combined side-channel and fault attack protected ASCON using threshold and triplication/majority. A recent brief described the implementation of ASCON as a peripheral of a RISC-V System on Chip (SoC)^14^, utilizing 180 nm CMOS technology and Artix-7 FPGA. In this setup, the ASCON core occupied 1424 look-up Tables (LUTs) in the FPGA and consumed 17.4 kGE (gate-equivalent) in 180 nm technology while achieving an energy efficiency of 417 Gbits/J at a frequency of 2 MHz.

### Limitations of previous implementations

- Most designs involve trade-offs between power, area, and speed.
- Many implementations rely on outdated CMOS technology nodes.
- ^10^ prioritizes area and power, resulting in a low frequency of 1 MHz and area of 2.57 KGE.
- ^12^ achieves 667 MHz but at the cost of 25.1 KGE area and 1.990 mW power; on Artix-7 FPGA, it runs at 244 MHz with 1045 FFs/1548 LUTs.
- ^13^ implements unprotected ASCON on Kintex-7 FPGA with 181.8 MHz frequency and high resource use of 1830 FFs/2809 LUTs.
- ^14^ achieves only 2 MHz at 180 nm technology with a significant area of 17.4 KGE.

### Our contribution

- A balanced design targeting low area, low power, and reasonable clock frequency.
- Implementing our design using advanced 16 nm FinFET technology to match modern trends.
- Reducing clock cycles by using a serialized architecture with a single S-box in the permutation phase.
- On the Lattice ICE40HX1K-STICK-EVN FPGA evaluation kit, achieving 200 MHz with only 418 FFs and 582 LUTs.
- On TSMC 16-nm technology, reaching 100 MHz with an area of 1.341 KGE and 0.254 mW power consumption.

### ASCON

ASCON utilizes a sponge construction framework in various ways. There are two modes for authenticated ASCON 128 (block size = 64) and ASCON 128a (block size = 128). Both take a 128-bit key (K) and nonce (N) and have a 320-bit state. Using the encryption method with two rounds of numbers (P6 and P12), an authenticated ciphertext CT is produced with a length identical to that of the plaintext PT and an authentication tag known as T, which has a size of k bits (128 bits). Two other ASCON modes for hashing employ SHA-3 (ASCON Hash and ASCON-Xof). SHA-3 consists of two phases: absorbing and squeezing. The hashing modes of ASCON have fixed rounds Pa, rate r (64-bit), and can be configured to a limited output of 265 bits to ensure security. All modes of ASCON share the process of permutation that is designed to be robust and adaptable to different types of attacks^6^.

**Fig. 2:**
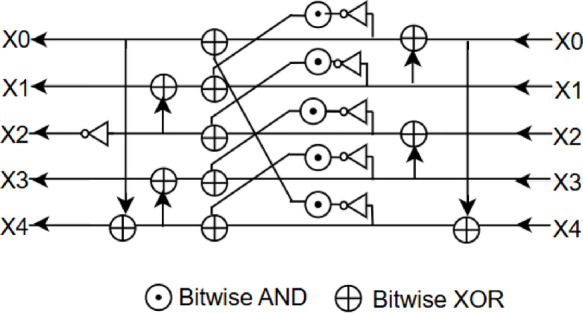
Substitution operation for 5-bit S-box layer.

**Fig. 3:**
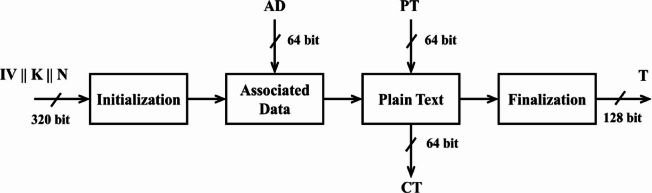
ASCON 128 authenticated mode.

**Fig. 4:**
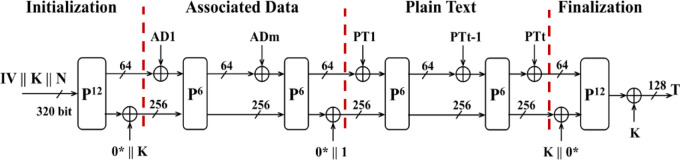
The encryption process stages.

A. Permutation The primary function of ASCON is to perform permutation through various rounds, specifically Pa and Pb. The number of rounds in Set P can be adjusted based on security parameters. After each round, the output is feedback, corresponding to the designated target round. The block or state (S) is divided into five 64-bit register words, denoted as Xi (S = X0 | X1 | X2 | X3 | X4), which store bits through three specific functions. These functions maintain a predetermined order across all rounds of the block. The series of layers is as follows: the addition, substitution, and linear diffusion layers, each contributing uniquely to ensuring encryption security^6^. The addition layer: adding a constant value according to the number of rounds to X2. The substitution layer, which introduces the nonlinear operation within the permutation, updates each bit slice (5 bits) formed by concatenating the five registers, with X0 as the MSB and X4 as the LSB. The S-box in ASCON is derived from an affine transformation of the χ mapping used in Keccak^15^ and can be implemented either as combinational logic, as shown in Fig. 2, or through a look-up table. The S-Box of ASCON provides resistance against side-channel attacks in combinational logic implementation. In addition, a low algebraic degree can include protection through masking or shared-based countermeasures. Linear diffusion layer: The third layer is responsible for diffusion on each 64 bits from X0 to X4 by applying a function that incorporates the XOR and the right rotation.
B. AEAD The authenticated ASCON family offers two modes, each supporting different plaintext sizes: ASCON 128 works with 64-bit blocks, while ASCON 128 supports 128-bit blocks. Each mode has a distinct number of permutation rounds, designated as Pa and Pb. In the authenticated mode of ASCON 128, the encryption process consists of four stages that build upon the previous block, ensuring a high level of security, as illustrated in Fig. 3. The initialization stage involves 320 bits, which includes a 128-bit input key (K), a unique 128-bit nonce (N) for each encryption, and an initialization vector (IV) specific to ASCON 128. These components undergo several rounds of permutation. An XOR operation is then performed on the capacity (C), calculated as 320 minus the rate (r), combined with the key, and zero-padding is applied on the left before moving on to the associated data. The second optional stage deals with associated data. Here, the input data is XORed with r bits, followed by zero-padding until the total size matches C, with a padding of 1 added. The central stage is the encryption stage, where the plaintext is padded to align with the size of the rate (r) before being encrypted to produce a 64-bit ciphertext (CT). The finalization stage generates the Tag (T), which is essential for verifying the decryption process at the receiving end. The mentioned stages are illustrated in Fig. 4.

### Hardware implementation architectures

Various hardware architectures have been developed for the ASCON core, each tailored to fulfill specific objectives within IoT applications.

A. Unrolled Implementation Also referred to as “fast ASCON,” this implementation prioritizes maximal data throughput while minimizing processing delay^10^. The fundamental principle of this approach involves executing all permutation rounds within a single clock cycle, which enhances performance by eliminating the need to register intermediate values. However, a significant drawback of this method is its requirement for a large silicon area, rendering it unsuitable for IoT applications with stringent area constraints.
B. Serial Implementation Conversely, the serial implementation, often referred to as “low area ASCON,” can be succinctly described as performing a “one-bit operation per cycle.” This serialized approach processes 5 × n-bit for an ASCON S-box in a single clock cycle, where n equals 2^*m*^ for *m* = {0, 1, 2, 3, 4}. As the size of the S-box is contingent upon n, increasing the number of bits processed per cycle results in a linear area increase, while the throughput experiences exponential growth^11^.
C. Round-Based Implementation The round-based implementation adopts a strategy that neither emphasizes high parallelism, as seen in fully unrolled designs, nor focuses solely on area optimization, akin to serial strategies. Instead, it provides a balanced trade-off between hardware area utilization and performance regarding throughput for ASCON. This implementation executes (*s*) ASCON permutations within a single clock cycle, where (*s*) can take values from the set {1, 2, 3. 12}^11^.

### Proposed hardware implementation

**Fig. 5:**
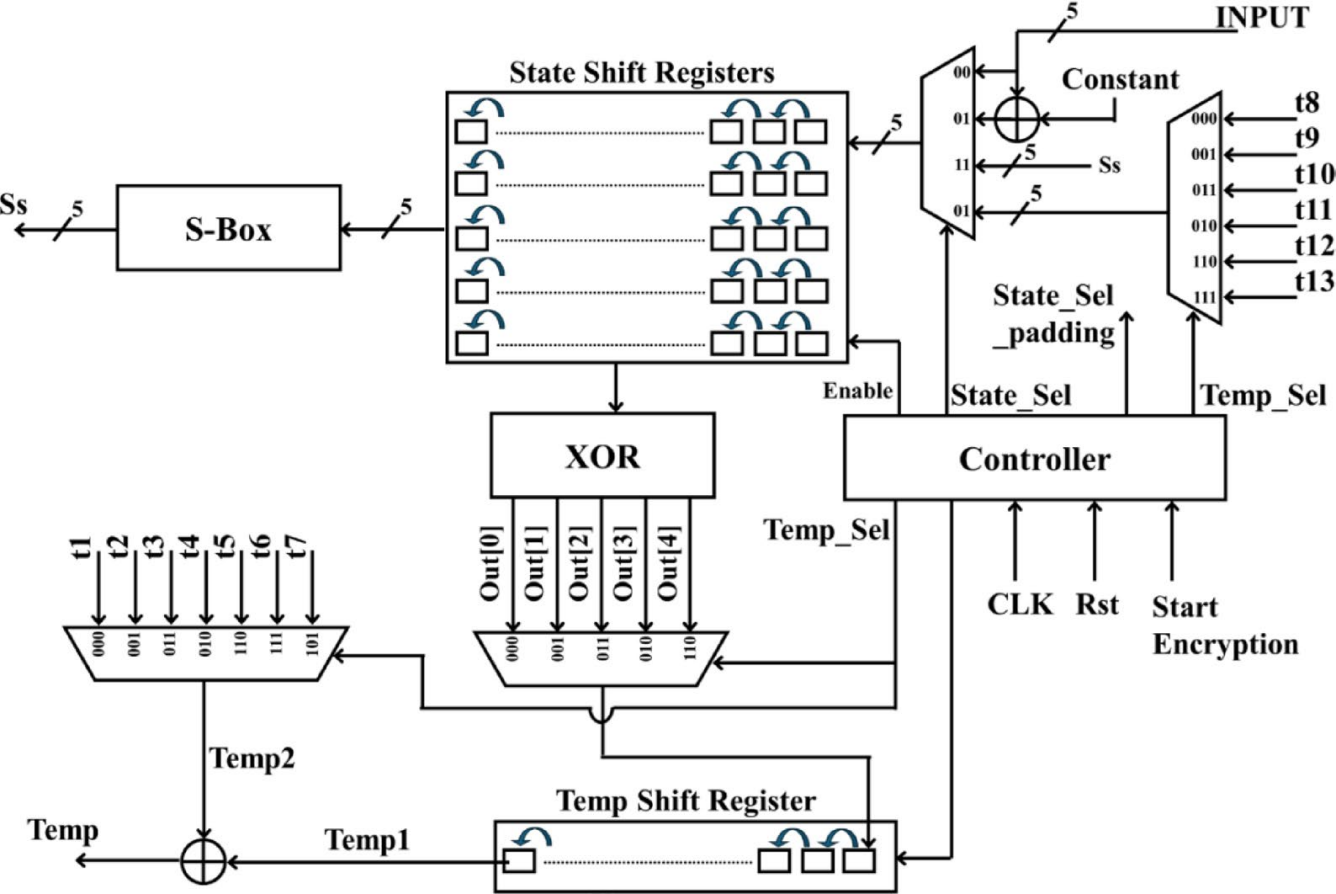
Proposed ASCON core architecture.

The presented architecture in Fig. 5 depicts an ASCON Core, which implements a serialized approach targeting low-area and low-power applications such as RFID tags. While ASCON is designed with inherently robust side-channel attacks, the presented work does not integrate dedicated countermeasures such as masking or redundancy. Previous work^10^ has proposed threshold implementation schemes to enhance side-channel resistance, albeit with a significant increase in area overhead. The primary focus of this design was to achieve the least possible area and power consumption while maintaining an acceptable throughput. This was achieved by employing a single 5-bit S-box (like the one-bit-per-cycle approach) instead of utilizing 64 parallel 5-bit S-boxes, thereby minimizing the overall area. The logic shown in Fig. 5 is supported by a set of control signals derived from conditional operations based on the state_sel_padding variable. These control temporary variables (t1–t13), which govern bit-level assignments, padding logic, and constant injection into the state during encryption rounds. For clarity and readability, these control signals are written in Verilog style. Each variable in the code corresponds to specific operations or datapaths shown in Fig. 5, e.g., t2–t6 handle dynamic input padding, while t7–t13 prepare values to be loaded into the State Shift Register and XORing block.

Control temporary variables:

t1 = 1’b0.

t2 = (state_sel_padding == 3’b10)? input_data[1]: 1’b0.

t3 = (state_sel_padding == 3’b10)? input_data[2]∶ 1’b0.

t4 = (state_sel_padding == 3’b10)? input_data[2]∶ 1’b0.

t5 = (state_sel_padding == 3’b01)? input_data[1]∶ (state_sel_padding == 3’b111)? input_data[1]: 1’b0.

t6 = (state_sel_padding == 3’b01)? input_data[2]∶ (state_sel_padding == 3’b011)? padd_with_one∶ (state_sel_padding == 3’b111)? input_data[2]∶ 1’b0.

t7 = 1’b0.

t8 = {Xs[0], 4’b0 }

t9 = {temp, Xs[1], 3’b0 }.

t10 = {1’b0, temp, Xs[2], 2’b0 }.

t11 = {2’b0, temp, Xs[3], 1’b0 }.

t12 = {2’b0, (temp ^constant), Xs[3], 1’b0 }

t13 = {3’b0, temp, Xs[4]}.

**Fig. 6:**
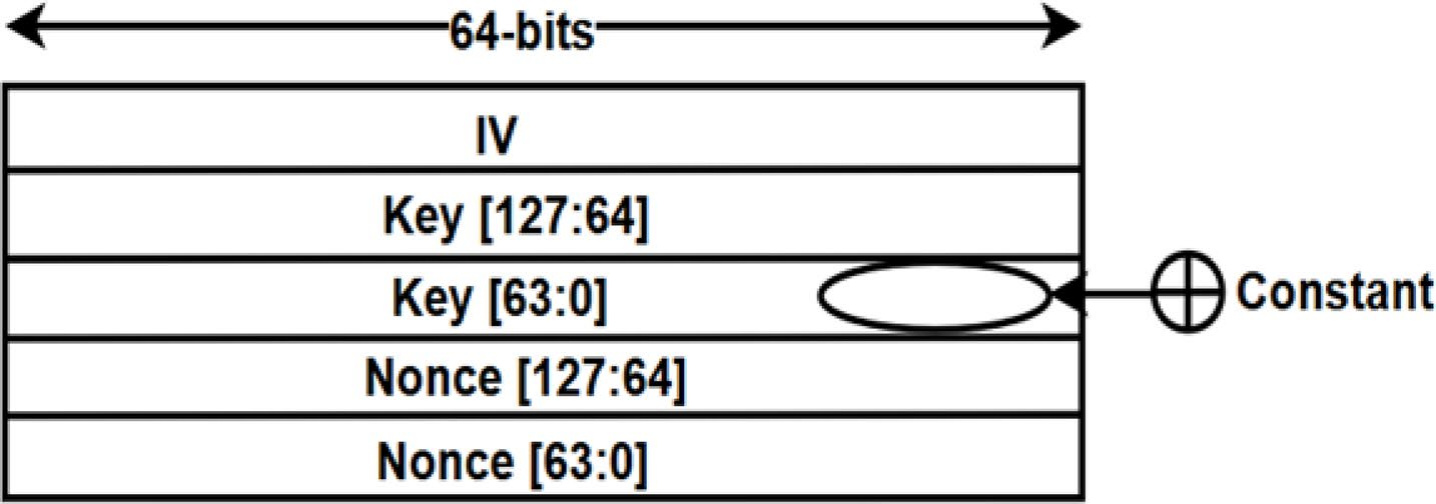
State shift registers.

The proposed data path consists of five parallel state shift registers, each 64 bits in size, with separate shift-enable signals. The initial data loading process consumes 64 cycles, where 56 cycles are dedicated to loading the input data (IV, Key, and Nonce), and the remaining 8 cycles are used to XOR the least significant 8 bits of the key with the corresponding round constant as shown in Fig. 6. This approach aims to improve the throughput by overlapping the key integration with the data loading, rather than waiting for the entire 64-cycle loading process and then performing the round constant operation.

After the initial data loading and round constant operation, the next step in the workflow is the substitution layer, which employs an S-box. To initiate the loading of the 5-bit S-box, the state shift registers are enabled and shifted in a bit-sliced manner, with 5 bits processed per clock cycle. Upon completion of the substitution layer, the results are stored in the least significant bits of the state shift registers. The inclusion of a multiplexer before the state shift registers facilitates the selection of the appropriate inputs, depending on the current operation.

**Fig. 7:**
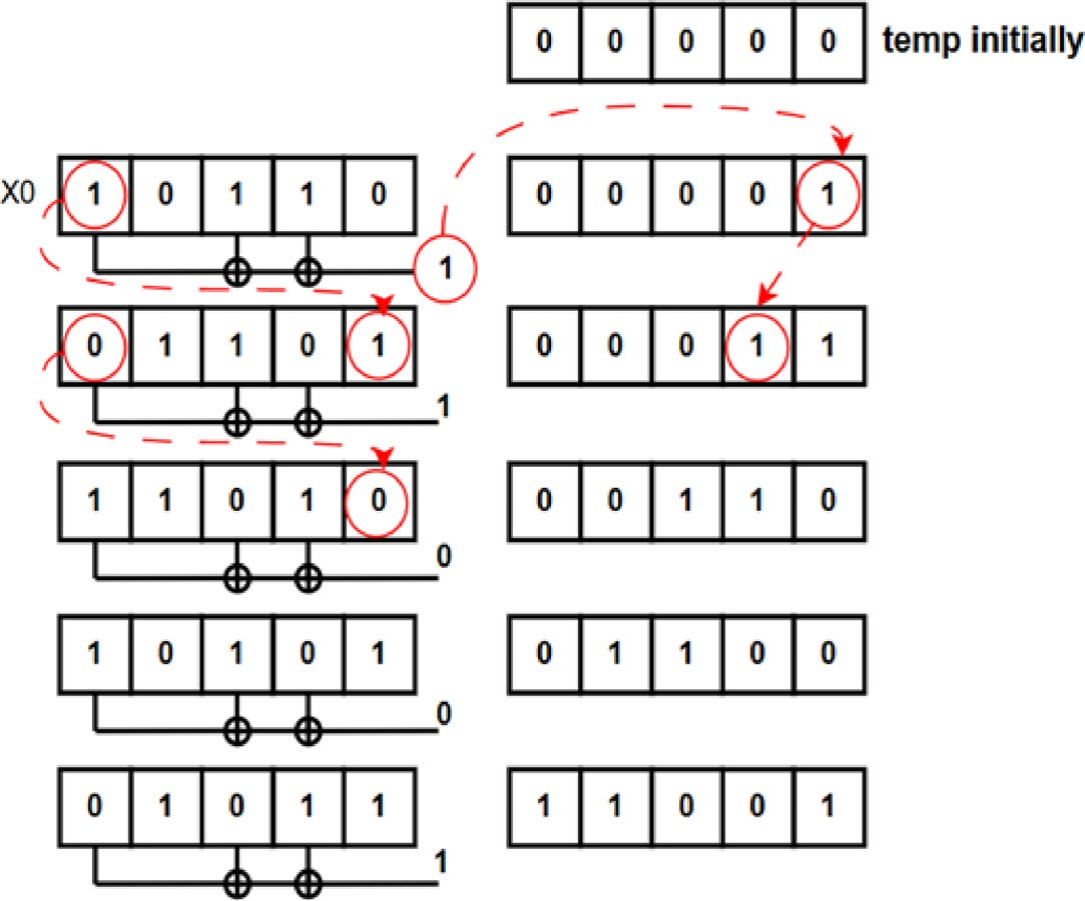
S-Box operation.

This S-box operation consumes an additional 64 cycles to complete. The subsequent step in the flow is the linear diffusion operation, as illustrated in the simplified example in Fig. 7.

$$\:\mathrm{x}\:=\:10110$$

$$\:\mathrm{y}\:=\:\mathrm{x}\:\oplus\:\:(\mathrm{x}\:\ggg 3)\:\oplus\:\:(\mathrm{x}\:\ggg 2)$$

The state shift registers in the proposed architecture are 64 bits in size (instead of 5 bits in the previous example), with each row updated separately. The linear diffusion operation is performed in a multi-step process. First, the updating of the temporary register is carried out over 64 cycles, processing one row (X0) at a time. Subsequently, the result stored in the temporary register is written back to the corresponding row (X0) in the next 64 cycles while simultaneously initiating the update of the temporary register for the next row (X1). This overlapping approach is repeated for the remaining rows (X1 through X4), resulting in 384 cycles for the complete linear diffusion operation (64 cycles for updating the temporary register, followed by 64 cycles for writing back each row).

Furthermore, during the write-back process for row X2, a simple XOR operation is performed on the least significant 8 bits with the next round constant, further improving the throughput.

The first round of the permutation process, encompassing the data loading, round constant integration, substitution, and linear diffusion operations, consumes 512 clock cycles. The subsequent rounds of permutation will require 448 clock cycles, as the input data (IV, Key, and nonce) is loaded only once at the beginning of the encryption process.

**Fig. 8:**
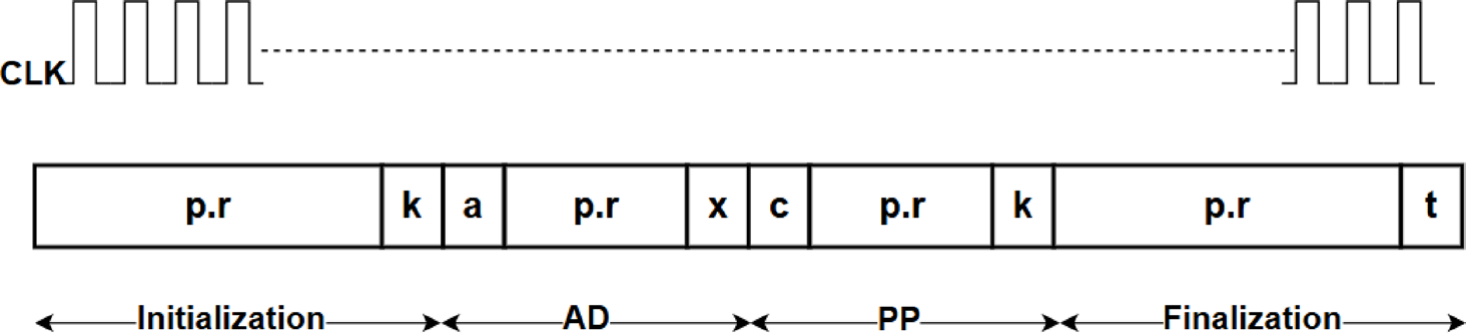
The whole encryption cycle.

As the ASCON encryption algorithm consists of 36 permutation rounds across four stages, the total latency of the proposed core is 16,192 clock cycles. This represents the time required to complete the full encryption of a single block with a single plaintext and a single associated data, as illustrated in Fig. 8.

### Hardware vs. software comparison

In evaluating lightweight cryptographic algorithms such as ASCON, both software and hardware platforms are commonly used^16,17^. Software implementations on general-purpose processors and microcontrollers offer greater flexibility, enabling algorithmic updates and portability. However, such implementations are constrained by instruction overhead, which results in high latency and energy consumption; for example, measured latencies of 164.3 cycles/byte on ARM Cortex-M4 processors^18,19^ and energy consumption reaching 5706 µJ/byte on Raspberry Pi Zero^20^. They also suffer from non-negligible idle power. These characteristics make pure software implementations unsuitable for always-on, ultra-low-power applications^17,20^. In contrast, dedicated hardware implementations, whether on FPGAs or ASICs, offer higher throughput, predictable latency, and significantly lower dynamic power consumption^10,16^. They can be tailored for various architectural goals. For example, software implementations running on RISC-V cores typically require hardware acceleration or instruction set extensions (ISEs) to meet performance and energy constraints. One such study reported 50× speedup for AEAD and 80× for hashing when ISE-based hardware was used over baseline software. Threshold implementations in hardware add robust side-channel protection at 3.7× area overhead, though direct performance comparisons remain challenging due to toolchain variations causing 30–60% FPGA throughput disparities^21^.

Finally, security is also a consideration; software implementations on microcontrollers are highly vulnerable to side-channel attacks, including Differential Power Analysis (DPA) and Correlation Power Analysis (CPA), often requiring just a few traces to break unprotected designs^10^. Nonetheless, as noted in^16,17^, direct cross-platform comparisons of metrics like power and throughput remain inherently unreliable due to variations in benchmarking methods, technology nodes, and toolchains. Therefore, our comparative analysis focuses on hardware implementations only, where the performance and resource metrics can be more fairly assessed.

**Fig. 9:**
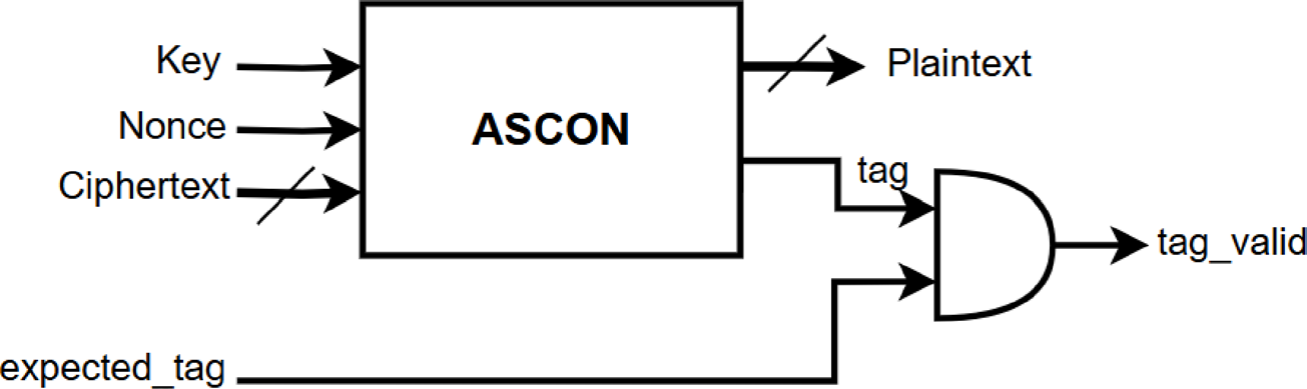
S-Box operation.

**Fig. 10:**
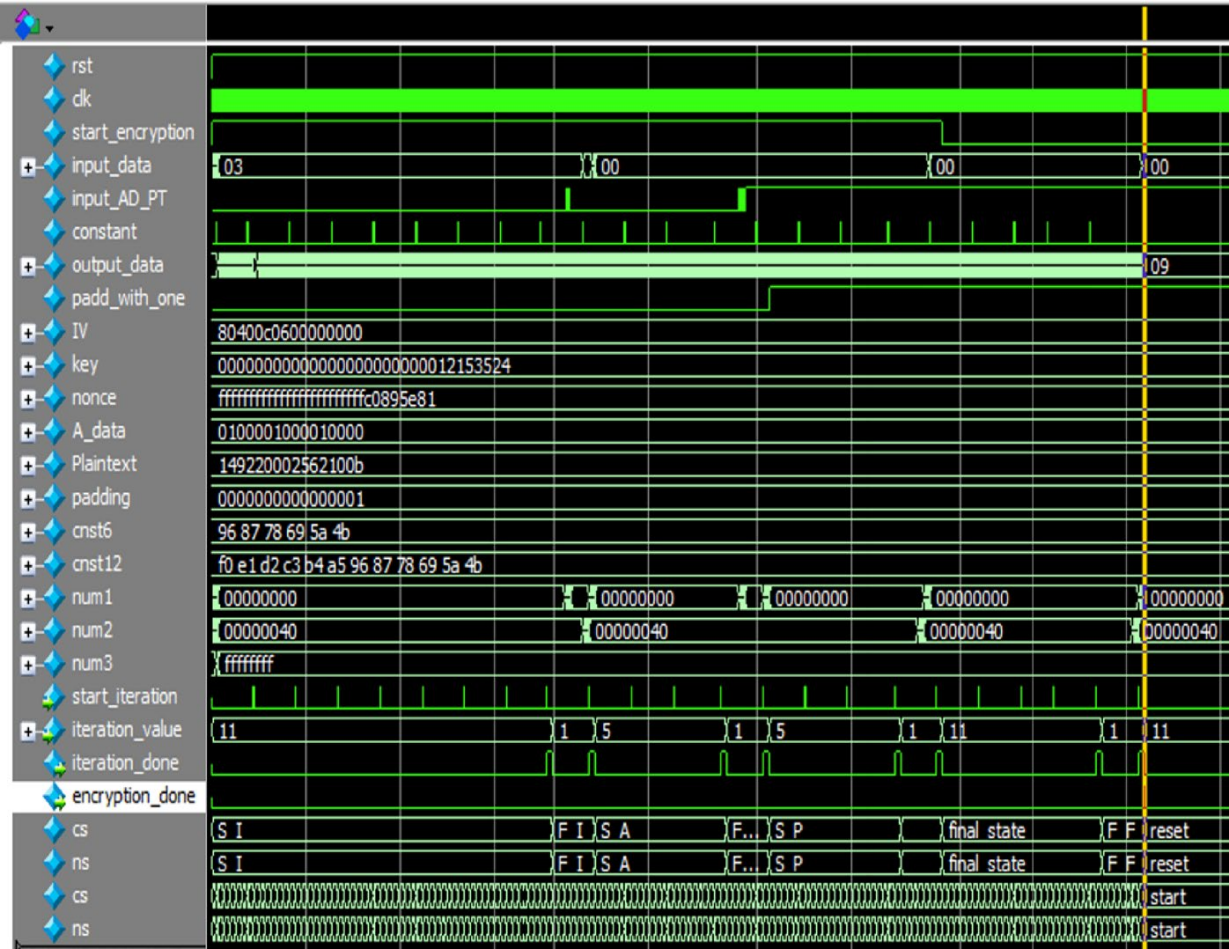
The whole encryption process.

**Fig. 11:**
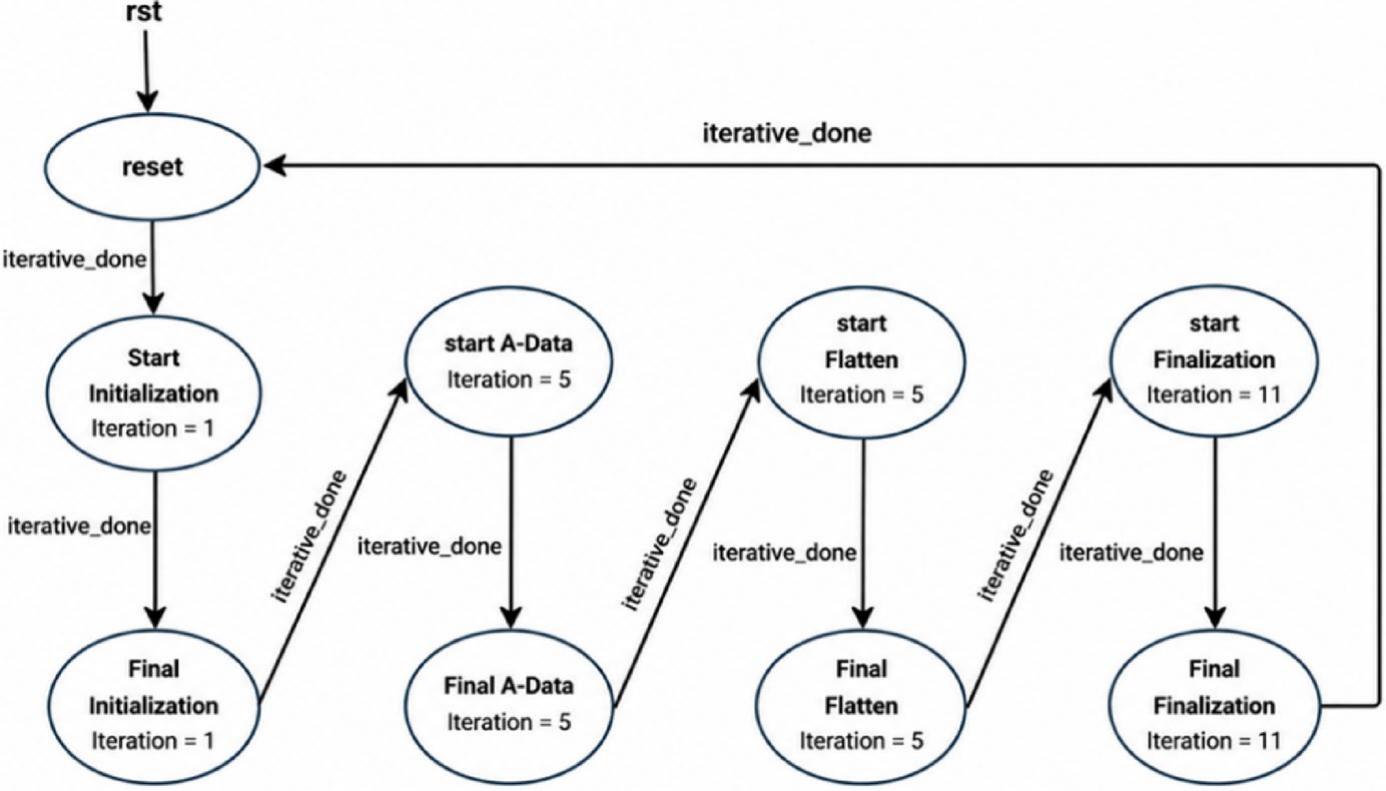
Finite state machine of the proposed ASCON.

## Results

### Simulation results

The testing methodology utilizes a random plaintext within the encryption block to generate the ciphertext and the tag. Subsequently, this ciphertext is utilized as the plaintext for the decryption block, which serves to regenerate the plaintext and the tag. The regenerated plaintext should be identical to the initial plaintext, and the tags should match. Additionally, a control flag is incorporated to ensure that the two tags correspond, as shown in Fig. 9. The encryption and decryption blocks depicted above are identical in structure. The sole distinction lies in the inputs utilized and the resultant outputs generated. In addition to the step of comparing the generated tag with the expected one.

The simulation of the entire encryption process is shown in Fig. 10, demonstrating the sequential flow of operations through the different stages. The proposed Finite State Machine (FSM), illustrated in Fig. 11, is designed with nine distinct states. These states collectively manage all 36-permutation rounds and ensure synchronized control across rounds while maintaining the serialized structure of the architecture.

### Implementation results

**Fig. 12:**
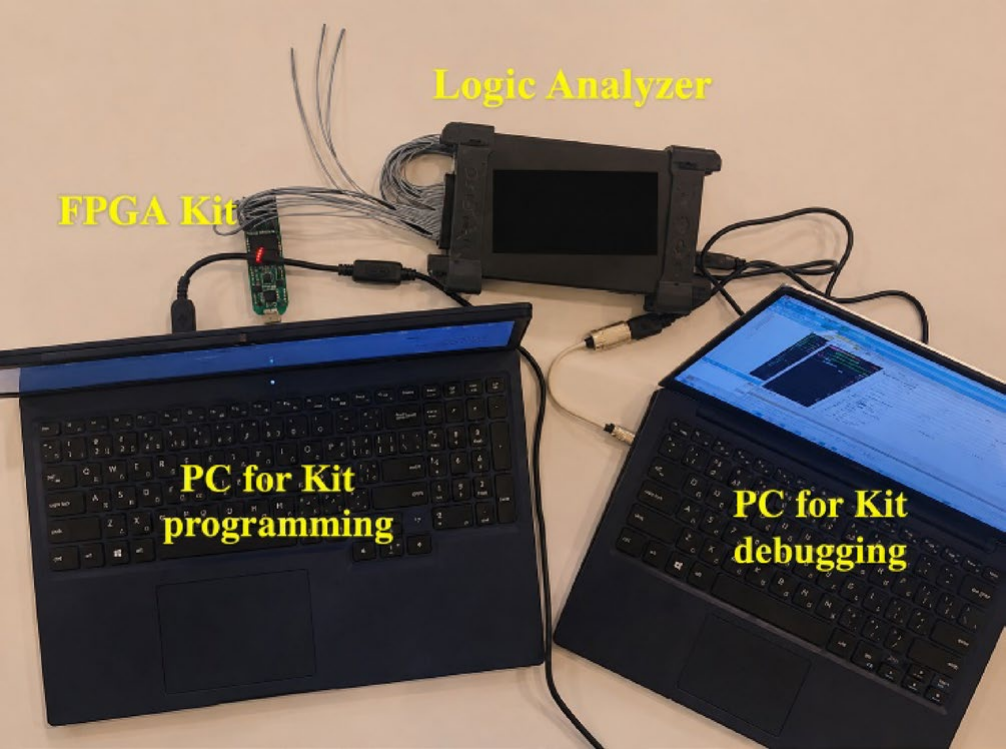
The hardware debugging setup using logic analyzer.

**Fig. 13:**
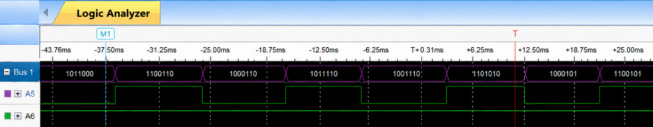
The logic analyzer waveform.

**Fig. 14:**
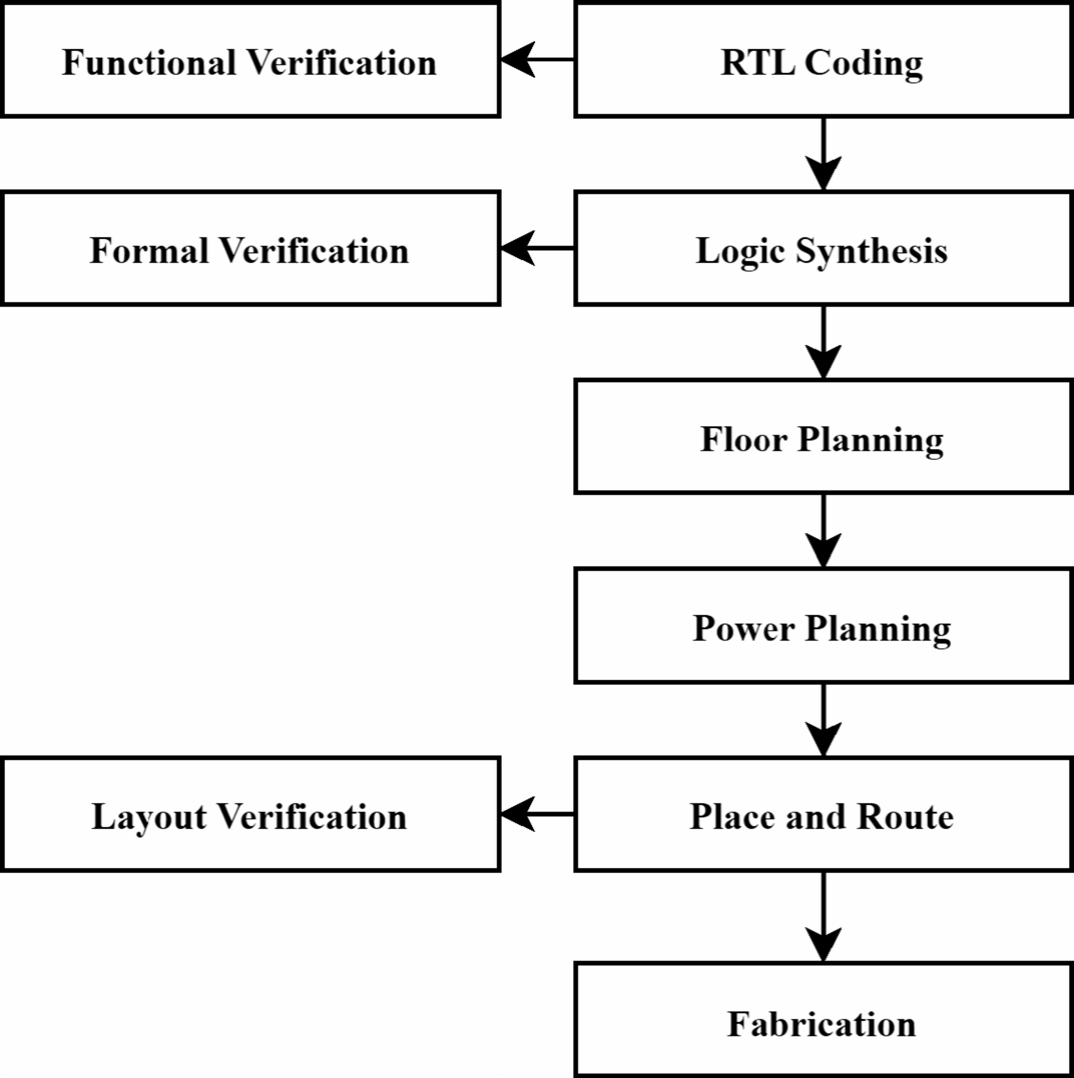
ASIC flow.

**Fig. 15:**
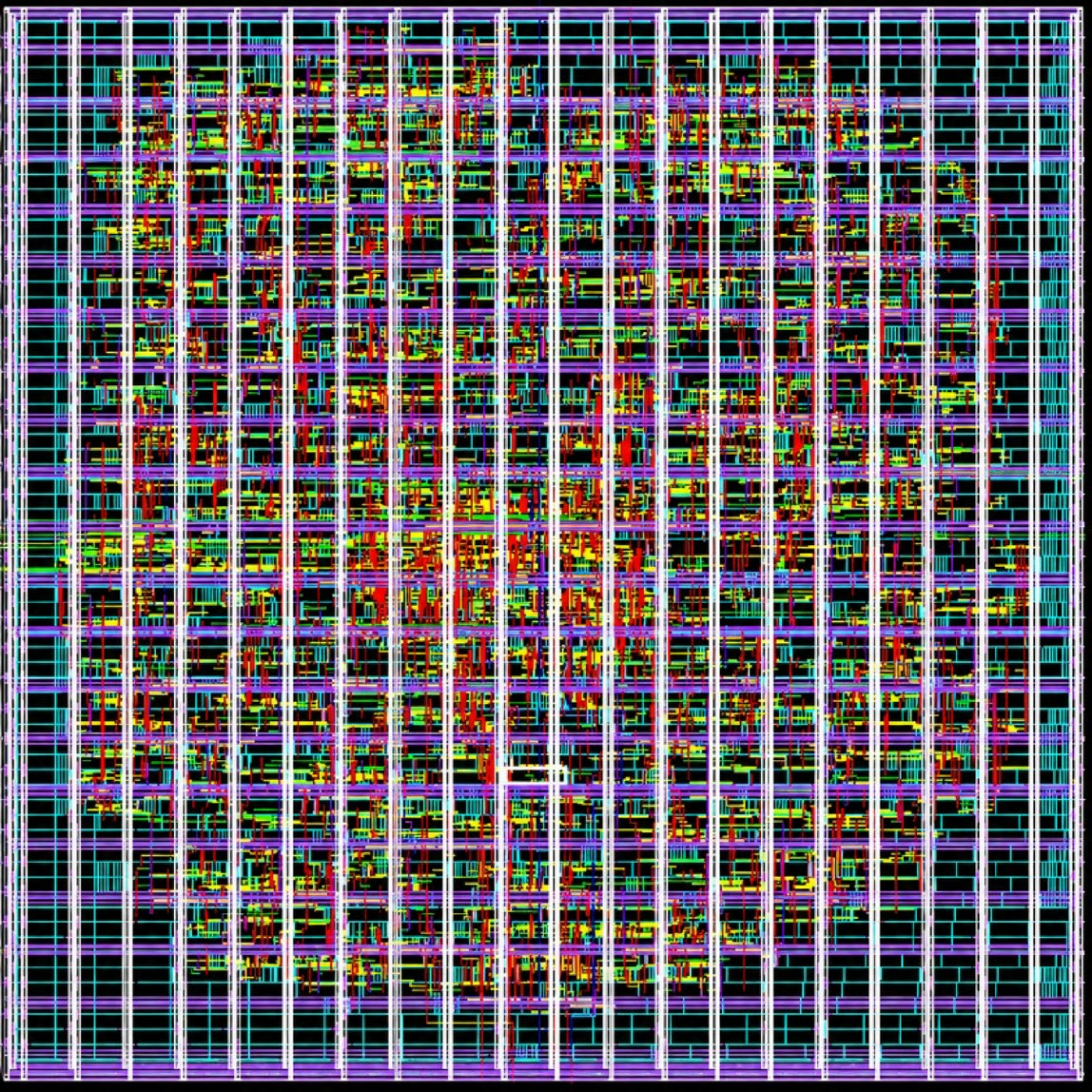
Layout view of proposed design in 16 nm FinFET.

A. FPGA The architecture presented in our study demonstrates a high degree of flexibility, allowing for implementation across a diverse range of FPGA families. The serial ASCON 128 is designed using the Verilog Hardware Description Language (HDL), specifically targeting the Lattice ICE40HX1K-STICK-EVN FPGA evaluation kit. The iCE40 devices are optimized for battery life and reduce power consumption, making them ideal for extremely low-power and always-on applications. For operation at the RFID frequency of 13.7 MHz, the design achieves resource utilization of 582 look-up tables (LUTs) and 418 flip-flops, with an estimated power consumption of 2.28 mW using the Lattice power estimator. Post-implementation timing analysis, performed using the Timing Analyzer, confirms that the design meets all timing constraints with no violations. The worst negative slack (WNS) is + 0.63 ns, the total negative slack (TNS) is 0 ns, and the achieved maximum operating frequency (Fmax) is 228.8 MHz. Power analysis is performed using the Lattice power estimator by assuming a uniform switching activity factor of 50%. At the target operating frequency of 200 MHz and voltage of 3.3 V, the estimated total power consumption is 27.75 mW. The design flow utilized iCEcube2 for synthesis and Lattice Diamond for implementation on the target device. The hardware debugging was performed using the logic analyzer to observe key signal transitions. A configuration module was added to the design to stimulate the input. The debugging setup is shown in Fig. 12, while Fig. 13 presents the captured waveform results from the logic analyzer, showcasing the 5-bit output sequences, along with the corresponding clock and reset signals. This setup enabled precise observation of timing relationships and functional correctness. Table 1 summarizes various implementations of lightweight cryptographic algorithms on FPGA platforms.
B. ASIC Most ASCON implementations are limited to 28 nm CMOS, a leading technology for high-end IoT devices. Our study aims to adapt the ASCON architecture for FinFET technology, addressing the challenges of shrinking process nodes over time. This transition demonstrates that the proposed architecture can meet the FinFET requirements for IoT applications at 16 nm. The proposed design targets a 16 nm TSMC educational version, developed with the support of commercial tools from Synopsys. The evaluation process involves using Design Compiler (DC) and IC Compiler II, which facilitates the progression from Register Transfer Level (RTL) to Graphic Data System II (GDSII), as shown in Fig. 14. The design can leverage support from IoT vendors or the research community, offering flexibility in implementation. Throughout the process, we applied area constraints on DC by setting area constraints to zero to achieve the minimum possible area while ensuring correct functionality at an operating frequency of 100 MHz. The gate-level netlist then undergoes formal verification to ensure design consistency and correctness concerning the RTL before proceeding to the layout stage, which begins with floor planning. The power planning stage employs a mesh strategy connected to power rings, ensuring robust power distribution across the chip. This is followed by the place-and-route stage, where the physical implementation of the design is performed. Afterward, layout verification is conducted, including Design Rule Checks (DRCs) to ensure compliance with manufacturing constraints and Layout Versus Schematic (LVS) checks to verify the consistency between the layout and the original schematic. The final layout satisfies all specified design constraints, achieving timing closure with a positive slack of 9.5 ns, and exhibits no setup or hold violations. This considerable timing margin indicates that the circuit has the potential to operate reliably at higher clock frequencies. Tools provide outcome results for area and power after the final stages of the 16 nm TSMC fast-fast process, with a temperature of 125 °C and a voltage of 0.88 V. The total power consumption for the design is comprised of leakage power 0.075 mW and dynamic power consumption 0.179 mW, resulting in an estimated total power consumption of 0.254 mW at a supply voltage of 0.88 V. The area report highlights that the netlist cell area is 802 μm², and the total cell area is 1509 μm², equivalent to 1.341 kGE, which includes both the netlist and physical components. Table 2 summarizes the ASIC results for power and resource utilization at a frequency of 100 MHz, comparing our work with other designs. Figure 15 presents the final layout of the ASCON algorithm in TSMC 16 nm FinFET technology.
C. Discussion The rate at which the design can process plaintext data into ciphertext refers to throughput in hardware cryptographic implementations, typically measured in bits per second (bps).

1 $$\:\mathrm{T}\mathrm{h}\mathrm{r}\mathrm{o}\mathrm{u}\mathrm{g}\mathrm{h}\mathrm{p}\mathrm{u}\mathrm{t}\:\left(\mathrm{b}\mathrm{p}\mathrm{s}\right)=\:\frac{\mathrm{N}\mathrm{u}\mathrm{m}\mathrm{b}\mathrm{e}\mathrm{r}\:\mathrm{o}\mathrm{f}\:\mathrm{b}\mathrm{i}\mathrm{t}\mathrm{s}\:\mathrm{p}\mathrm{r}\mathrm{o}\mathrm{c}\mathrm{e}\mathrm{s}\mathrm{s}\mathrm{d}\:\:\mathrm{*}\:\mathrm{C}\mathrm{l}\mathrm{o}\mathrm{c}\mathrm{k}\:\mathrm{F}\mathrm{r}\mathrm{e}\mathrm{q}\mathrm{u}\mathrm{e}\mathrm{n}\mathrm{c}\mathrm{y}}{\mathrm{L}\mathrm{a}\mathrm{t}\mathrm{e}\mathrm{n}\mathrm{c}\mathrm{y}\left(\mathrm{i}\mathrm{n}\:\mathrm{c}\mathrm{l}\mathrm{o}\mathrm{c}\mathrm{k}\:\mathrm{c}\mathrm{y}\mathrm{c}\mathrm{l}\mathrm{e}\mathrm{s}\right)\:}$$

In Eq. (1), the block size corresponds to the number of bits processed per encryption block—also known as the rate. For ASCON-128, the rate is 64 bits: each permutation processes 64 bits of plaintext and produces 64 bits of ciphertext per block. The associated data, however, is processed separately at rate of 64 bits, contributing to the authenticated encryption without expanding the ciphertext size. The clock frequency in the equation refers to the operating speed of the hardware, while the number of clock cycles indicates the cycles required to complete one full encryption operation. The total latency is 16,192 clock cycles, consisting of 5,440 cycles for initialization, 2,688 cycles for associated data processing, 2,688 cycles for plaintext encryption, and 5,376 cycles for finalization. The proposed design achieves an energy efficiency of 0.106 nJ/bit and a throughput of 2.381 Mbps for plaintext encryption when operating at a frequency of 100 MHz.

Furthermore, compared to the previous contribution in^10^, which reports 512 cycles/permutation operation in their low-area ASCON design, the proposed architecture achieves a 12.5% reduction, requiring only 448 clock cycles. While both designs prioritize area and power efficiency, this optimization in cycle count directly contributes to lower latency and higher throughput under the same operating conditions. Tables 1 and 2 present a comparative analysis of the proposed ASCON-128 design against several state-of-the-art implementations on FPGA and ASIC platforms, respectively. Unlike most prior works^12,13,16^, which adopt parallel architectures optimized for high-throughput, our novel design employs a serialized approach, trading off peak throughput for superior area and power efficiency – a critical advantage for ultra-constrained environments such as RFID tags or passive IoT devices.

In FPGA implementation, our architecture operates at 13.7 MHz (aligned with RFID standards) while utilizing only 582 LUTs and 418 Flip-Flops, achieving an ultra-low power consumption of 2.27 mW, which outperforms high-throughput designs in terms of efficiency. For instance^16^, consumes 222 mW at 317 MHz with 917 FFs, making it too power-hungry for energy-constrained applications.

In the ASIC domain, prior implementations either target older CMOS technologies (e.g., 90 nm^10^, 130 nm^13^ or focus on throughput-heavy parallel architectures^12,22,23^. In contrast, our work presents the first serialized implementation of ASCON-128 in a 16 nm TSMC FinFET node, which shows a significant area reduction (1.341 kGE), 49% smaller than^10^, and ultra-low dynamic power (0.254 mW at 100 MHz). While^10^ reports 0.015 mW at 1 MHz, but lacks full encryption stage implementation details and uses outdated 90 nm technology, making direct comparison unreliable. Crucially, no prior work employs a serial datapath or targets the same technology node and constraints. Thus, although our design sacrifices peak throughput, its unmatched area and power efficiency make it ideal for secure, resource-limited devices (e.g., IoT sensors, passive tags). The presented design does not currently integrate dedicated countermeasures such as masking or redundancy. Future efforts will focus on incorporating these security-enhancing techniques to improve resistance against side-channel and fault injection attacks.

**Table 1:** The comparison between different LWC designs on FPGA.

Design	FPGA					
	Chip Family	Operating Freq. (MHz)	Resource	Power (mW)	Throughput (Mbps)	Energy (nJ/bit)
This work	Lattice iCE40	200	418 FFs/582 LUTs	27.74	4.761	5.827
	Lattice iCE40	13.7	418 FFs/582 LUTs	2.27	0.326	6.963
RECO-HCON^12^	Artix-7	244	1045 FFs/1548 LUTs	-	5926	-
Unprotected ASCON^13^	Kintex-7	181.8	1830 FFs/2809 LUTs	-	-	-
ASCON^16^	Artix-7	317	912 FFs/1756 LUTs	222	376	0.590

**Table 2:** The comparison between different LWC ASIC designs.

Design	ASIC					
	Tech. Node	Operating Freq. (MHz)	Resource	Power (mW)	Throughput (Mbps)	Energy (nJ/bit)
This Work	16 nm TSMC FinFET	100	1.341 (kGE)	0.254	2.381	0.106
ASCON^10^	90 nm CMOS	1	2.57(kGE)	0.015	14	0.001
RECO-HCON^12^	28/32nm CMOS	667	25.1(kGE)	1.990	5926	0.0003
Unprotected ASCON^13^	130 nm STM CMOS	-	184,289 μm²	1.34	-	-
GIFT^22^	90 nm CMOS	10	1.35(kGE)	0.074	1249	0.00006
Grain^23^	65 nm CMOS	2300	2.79(kGE)	0.240	1150	0.0002

## Conclusion

This work presents a serialized hardware implementation of the ASCON-128 algorithm tailored for ultra-constrained environments such as RFID tags, embedded systems, and IoT devices. The proposed design adopts a low-area, low-power architecture while maintaining functional performance, with implementations on FPGA and ASIC platforms. On FPGA, the architecture demonstrated compatibility with ultra-low-power platforms such as the ICE40HX1K-STICK-EVN FPGA evaluation kit, achieving a maximum operating frequency of 228.8 MHz. It exhibited minimal resource utilization, only 582 LUTs and 418 flip-flops on the iCE40, and maintained power consumption as low as 2.28 mW at 13.7 MHz. For ASIC implementation, the design was successfully implemented using 16 nm TSMC FinFET technology, consuming only 0.254 mW with an area of 1.341 kGE, representing a novel contribution, as prior ASCON implementations have been limited to older CMOS nodes. A key architectural optimization includes reducing the number of clock cycles per permutation operation from 512 to 448 compared to previous serialized designs. This reduction improves both latency and throughput efficiency. Future work may focus on exploring pipelining techniques to enhance throughput, allowing faster data processing without compromising the design’s efficiency. Furthermore, integrating multi-mode capabilities, including support for ASCON-128a and hashing variants, would significantly broaden the applicability of the design across various use cases in the IoT landscape. Overall, this work provides a validated, energy-efficient, and scalable hardware solution for authenticated encryption.

## Data Availability

All data generated or analyzed during this study are included in this article. However, all datasets used during the current study are also available from the corresponding author on reasonable request.
